# ZnCr_2_O_4_ Inclusions in ZnO Matrix Investigated by Probe-Corrected STEM-EELS

**DOI:** 10.3390/ma12060888

**Published:** 2019-03-16

**Authors:** Wei Zhan, Andrey Yurievich Kosinskiy, Lasse Vines, Klaus Magnus Johansen, Patricia Almeida Carvalho, Øystein Prytz

**Affiliations:** 1 Department of Physics, Centre for Materials Science and Nanotechnology, University of Oslo, N-0316 Oslo, Norway; zhanwei2009@163.com (W.Z.); andrey.kosinskiy@ntnu.no (A.Y.K.); lasse.vines@fys.uio.no (L.V.); k.m.h.johansen@fys.uio.no (K.M.J.); 2 SINTEF Materials and Chemistry, NO-0314 Oslo, Norway; patricia.carvalho@sintef.no

**Keywords:** interface strain, band gap, scanning transmission electron microscopy (STEM), electron energy-loss spectroscopy (EELS), ZnO, ZnCr_2_O_4_

## Abstract

The ZnCr_2_O_4_/ZnO materials system has a wide range of potential applications, for example, as a photocatalytic material for waste-water treatment and gas sensing. In this study, probe-corrected high-resolution scanning transmission electron microscopy and geometric phase analysis were utilized to study the dislocation structure and strain distribution at the interface between zinc oxide (ZnO) and embedded zinc chromium oxide (ZnCr_2_O_4_) particles. Ball-milled and dry-pressed ZnO and chromium oxide (α-Cr_2_O_3_) powder formed ZnCr_2_O_4_ inclusions in ZnO with size ~400 nm, where the interface properties depended on the interface orientation. In particular, sharp interfaces were observed for ZnO [2$\bar{1}\bar{1}$3]/ZnCr_2_O_4_ [1$\bar{1}$0] orientations, while ZnO [1$\bar{2}$10]/ZnCr_2_O_4_ [112] orientations revealed an interface over several atomic layers, with a high density of dislocations. Further, monochromated electron energy-loss spectroscopy was employed to map the optical band gap of ZnCr_2_O_4_ nanoparticles in the ZnO matrix and their interface, where the average band gap of ZnCr_2_O_4_ nanoparticles was measured to be 3.84 ± 0.03 eV, in contrast to 3.22 ± 0.01 eV for the ZnO matrix.

## 1. Introduction

Oxides with spinel structures are scientifically and technologically interesting, with a wide range of applications. Zinc chromite (ZnCr_2_O_4_) is a spinel with a direct and wide band gap of ~3.8 eV [1,2], and has been proposed as a material for gas and humidity sensing [3,4,5], as a depollution catalytic material for reactions like the oxidation of hydrocarbons and the oxidative dehydrogenation of hydrocarbons [4,6,7], as a photocatalyst [8,9], and in magnetic applications [10]. Moreover, combined with another wide band gap oxide such as zinc oxide (ZnO), it has been proposed as a catalyst for the dehydrogenative condensation of glycerol for improving the economics of biodiesel production [11], as a photocatalytic material for wastewater treatment [12], and in gas sensing [13]. In such mixed oxide or heterojunction applications, the ZnO/ZnCr_2_O_4_ interfaces are of utmost importance. That is, a defect- and strain-free interface between ZnO and ZnCr_2_O_4_ is a prerequisite. For example, density functional theory calculations have shown that the (100) surface of the ZnCr_2_O_4_ spinel has a favorable oxygen vacancy formation energy [14]. Thus, in order to find the optimal heterostructure interface, a large range of orientations must be studied. However, investigating the strain across a range of different thin film heterostructures having different interface orientations can be exhaustive. An alternative approach is to utilize, for example, a ZnCr_2_O_4_ inclusion embedded in a ZnO matrix where the different interface orientations are readily available. This approach can be realized by, for example, ball-milling, dry pressing, and sintering at high temperatures [15].

To study the strain across heterojunction interfaces, one can adopt high-angle annular dark-field (HAADF)-scanning transmission electron microscopy (STEM), which makes use of incoherently and elastically scattered electrons to obtain images with high spatial resolution and atomic-number (Z) contrast [16,17,18]. Characterization of strain associated with extended defects has been further boosted by the successful application of geometric phase analysis (GPA) in probe-corrected high-resolution HAADF images [19,20,21].

In addition, as the size of a semiconductor particle reduces down to nanoscale (i.e., below 10 nm), its band gap increases as a result of quantum confinement effects, providing intriguing optical and electronic properties [22,23,24]. Previously, UV-Visible reflectance spectroscopy was employed to analyze the average band gap of ZnCr_2_O_4_ nanoparticles [2]. However, conventional techniques such as photoluminescence (PL), cathodoluminescence (CL), optical absorption, and UV-Visible reflectance spectroscopy have difficulty in measuring the band gap of one single nanoparticle because of poor spatial resolution. The rapid advances in the field of monochromatic electron energy-loss spectroscopy (EELS) during the past decade have attracted wide interest in EELS band structure determination [25,26,27]. By means of monochromated EELS in combination with probe-corrected STEM, recent research has made band gap mapping on the nanometer scale possible [28], opening the possibility of observing quantum confinement as the size of the particles are reduced. However, due to experimental and data process complexities, to date few EELS investigations have been carried out which measured the band gap of single embedded nanoparticles.

In this work, probe-corrected atomic-resolution STEM imaging coupled with GPA was utilized to characterize the structure and strain at ZnO/ZnCr_2_O_4_ interfaces. Furthermore, core-loss EELS revealed a chemically pure ZnCr_2_O_4_ phase. In addition, making use of monochromated low-loss EELS, the absorption onset originating from the ZnCr_2_O_4_ band gap was extracted, and a two-dimensional mapping of single particles with a diameter around 500 nm was demonstrated. These results also provide the necessary foundation to study smaller particles in the future, for the potential direct observation of quantum confinement leading to an increase in the band gap.

## 2. Experimental Methods

The specimen of ZnO with ZnCr_2_O_4_ nano-inclusion was prepared as follows. ZnO powder was mixed with α-Cr_2_O_3_ powder (ZnO: Sigma-Aldrich, St. Louis, MI, USA, 99.999% 6.10 g; α-Cr_2_O_3_: Sigma-Aldrich, 99.95%, 0.76 g), and the molar ratio ZnO:α-Cr_2_O_3_ was 15:1. The mixed powders were ball-milled for 3 h, then uniaxially dry-pressed at a pressure of 2805 MPa, and sintered at 1350 °C for 24 h in air, with a temperature increase and decrease rate of 450 °C/h.

Parts of the sintered specimen were ground into powder for X-ray diffraction (XRD) measurement. Silicon standard ASM 640d (NIST, Gaithersburg, MD, USA) powder was mixed with the specimen as a reference for calibration of the peak positions. The XRD experiment was performed on Bruker ASX D8 Discover (Billerica, MA, USA) with a Cu Kα_1_ source. Unit cell parameters were determined via Rietveld-software TOPAS Version 4.2.

The specimen for STEM studies was prepared from the sintered material by mechanical cutting, grinding/polishing, and finally ion beam thinning. Immediately before carrying out the STEM experiments, the specimen was cleaned in a Fischione Model 1020 plasma chamber. All the images were acquired by a probe-corrected and monochromated FEI Titan G2 60-300 (Hillsboro, OR, USA) equipped with four FEI super-X Energy-dispersive X-ray spectroscopy (EDX) detectors. The spatial resolution was approximately 0.8 Å for STEM imaging at a high tension of 300 kV. Energy-dispersive X-ray spectroscopy (EDX) was utilized to map the various elements. For simultaneous HAADF (Fischione Model 3000, Export, PA, USA) and annular bright field (ABF) observations, the probe convergence angle was set at 22 mrad, while the collection angles were 98.7–200 mrad and 10.6–21.5 mrad, respectively. To directly address the atomic structure, STEM image simulations were carried out using the QSTEM program [29] based on the multi-slice method.

All EELS experiments were performed using a Gatan GIF Quantum 965 (Gatan Inc., Pleasanton, CA, USA). For energy-loss near-edge fine structure (ELNES) analysis the energy resolution was approximately 0.8 eV as determined by the full width at half maximum of the zero-loss peak (ZLP). Monochromated EELS were conducted to measure the band gap of ZnCr_2_O_4_ nanoparticles two-dimensionally. In order to limit the Cherenkov radiation effect, an accelerating voltage of 60 kV was utilized: the spectral resolution was approximately 0.15 eV. The TEM specimen was finally thinned to be about 30 nm, further reducing the Cherenkov retardation losses efficiently. The theoretical spatial resolution of EELS is defined by the inelastic delocalization length ($L_{50}$). According to Equations (4)–(7) reported by R.F. Egerton [30], for 60 keV incident electrons, EELS spatial resolutions in measuring band gaps of ZnO (~3.3 eV) and ZnCr_2_O_4_ (~3.8 eV) are about 5.4 nm and 4.9 nm, respectively, which is highly suitable to characterize the band gap of the ZnCr_2_O_4_ particles in this work. The band gap extraction procedures used here were the same as those described previously [28,31,32]. In brief, a power-law model was employed to remove the background from the ZLP. The fitting range for background subtraction was 2.4–2.9 eV. Then, the direct band gap value was extracted by performing a curve fitting based on the parabolic band approximation.

Strain field across ZnO/ZnCr_2_O_4_ interfaces were evaluated with a GPA script developed for the DigitalMicrograph package (Gatan Inc., Pleasanton, CA, USA) [19,20,21,33,34,35]. This Fourier-space image processing technique translates the displacements affecting specific periodicities in the image into phase shifts of the average or reference passband, which are then mapped across the image and used to calculate the local strain. Real-space peak detection is not required, and there is no need to assign appropriate sublattices, therefore robust and straightforward two-dimensional strain mappings can be obtained from atom-resolved images of interfaces provided some degree of coherency.

## 3. Results and Discussion

**Figure 1:**
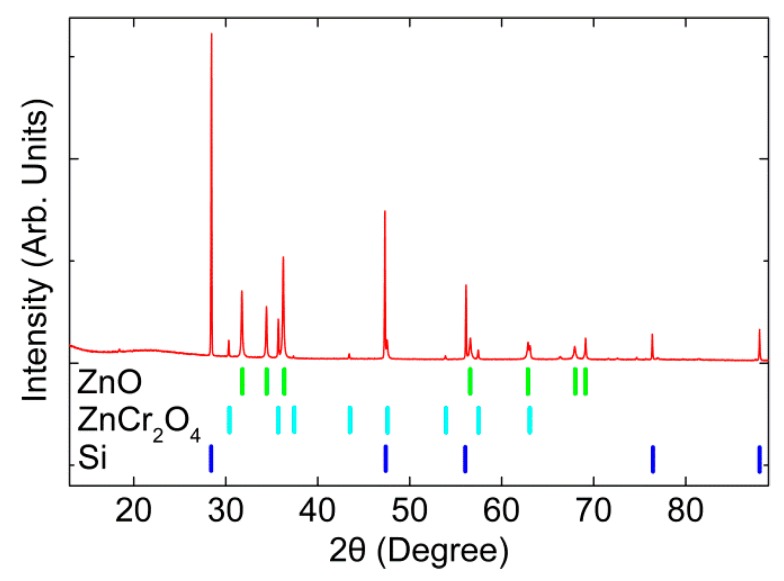
XRD of Cr2O3-alloyed ZnO with nano ZnCr_2_O_4_ inclusion. The Si-peaks were used to calibrate the diffractogram.

XRD measurement of the sample described in the experimental section is shown in Figure 1. Two crystalline phases were identified: a wurtzite ZnO and a spinel ZnCr_2_O_4_ phase. Except for the Si pattern used as a reference, no other phases were observed. Furthermore, the peaks were sharp and narrow, indicating high crystalline quality. The ZnO pattern was characteristic of the non-centrosymmetric wurtzite structure, while ZnCr_2_O_4_ crystallized in the antiferromagnetic spinel structure. The refined cell parameters of the ZnO and ZnCr_2_O_4_ can be found in Tables S1 and S2 (Supplementary Materials), respectively. The atomically resolved STEM images in Figure S1 also confirm the existence of these two phases. Experimentally determined unit cell dimensions for the ZnO phase were *a* = *b* = 3.2505(4) Å and *c* = 5.2059(6) Å. There was a slight shift from the original unalloyed ZnO with *a* = *b* = 3.2555(2) Å and *c* = 5.2152(3) Å [36]. Note that the ionic radius of Cr^3+^ (0.064 nm) was smaller than that of Zn^2+^ (0.074 nm) in the tetrahedral coordination. The peak shift and decreased lattice constant *c* confirmed the successful incorporation of Cr^3+^ ions into the ZnO host lattice, substituting for the Zn^2+^ sites [37]. The concentration of Cr was approximately 0.8 at% based on the EDX quantification in this work.

**Figure 2:**
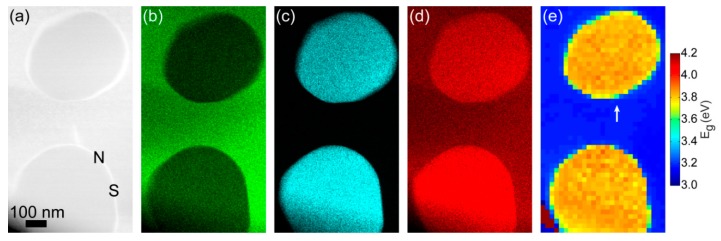
(**a**) Annular dark field (ADF) image of ZnO matrix and ZnCr_2_O_4_ nanoparticles; EDX maps of (**b**) Zn, (**c**) Cr, (**d**) O; (**e**) Band gap map.

The inclusion of ZnCr_2_O_4_ particles in a matrix of ZnO can be seen in the annular dark field (ADF) image in Figure 2a and in the corresponding EDX maps of Zn, Cr, and O in Figure 2b–d, respectively. The sizes of the two particles in Figure 2 were 450 nm (upper) and 500 nm (bottom) in diameter, respectively. EDX mapping such as those in Figure 2b–d confirmed that the diameter of ZnCr_2_O_4_ nanoparticles was approximately 100–500 nm. Taking advantage of probe-corrected and monochromated STEM-EELS, we performed two-dimensional band gap measurements of ZnCr_2_O_4_ nanoparticles in ZnO matrix. Figure 2e displays the band gap mapping outcome, which was extracted from the same region as elemental EDX maps. The size of this band gap map was approximately 592 nm (width) × 1073 nm (height). The yellow and blue colors represent high and low band gaps, respectively. Interestingly, the contrast shift observed in the ADF and EDX images was closely followed by a change in the absorption onset from the increased band gap of ZnCr_2_O_4_ as compared to ZnO. With a fitting range 2.4–2.9 eVfor background subtraction, the average band gap of ZnO was found to be approximately 3.22 eV, with standard deviation of 0.01 eVas extracted from 80 pixels. This is consistent with previous measurements [28,31]. The average band gap of ZnCr_2_O_4_ particles was measured to be 3.84 ± 0.03 eVs, being in line with existing reports for bulk values [1,2]. These results suggest that STEM-EELS produced reliable band gap measurements of single embedded particles. Furthermore, both sharp and non-sharp interfaces were available by considering different edges of the ZnCr_2_O_4_ nanoparticle—see the labels “*S*” and “*N*”, respectively (Figure 2a). In the EELS core loss region across a sharp interface (see Figure 4), the delocalization effect was reduced, and an improved lateral resolution could be obtained as compared to the low-loss EELS, but also as compared to the secondary X-ray emission process probed in EDX.

**Figure 3:**
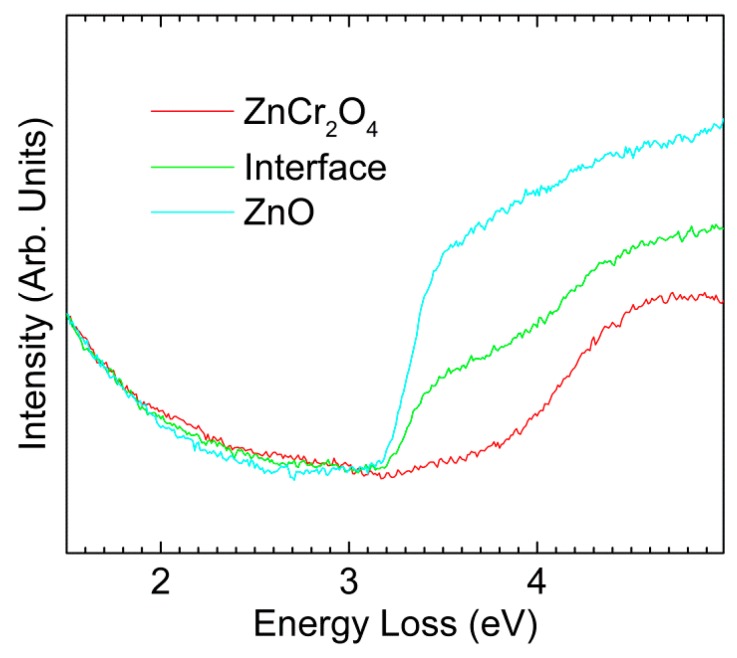
EELS spectra of ZnCr_2_O_4_, ZnO and the interface position.

Figure 3 shows low-loss EELS spectra extracted from the spectrum image used in Figure 2e. The spectra were taken from one pixel at the interface, and the two immediately adjacent pixels—one on the ZnCr_2_O_4_ side and one on the ZnO side. The three neighbor spectra positions are indicated by the white arrow in Figure 2e. As predicted, it can be clearly seen from the onsets that the band gap of ZnCr_2_O_4_ was higher than that of ZnO. The size of each pixel is about 22 nm × 22 nm, which means that for both materials $L_{50}$ was shorter than the size of each pixel. Thus, the ineleastic delocalization was limited to the interface pixel, where the spectrum revealed a mixture of the ZnO and ZnCr_2_O_4_ due to the inelastic delocalization of the bandgap signal [30].

**Figure 4:**
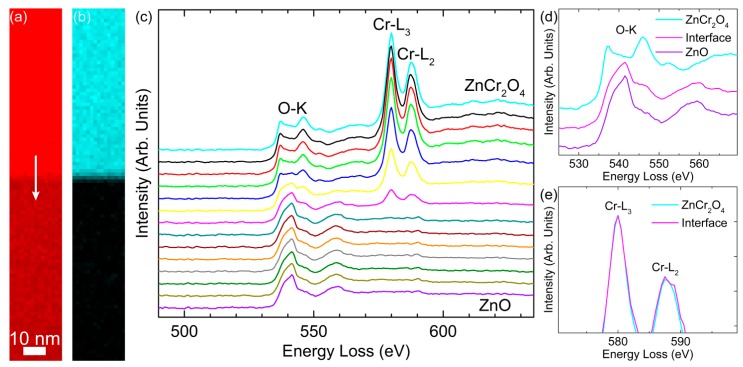
Core-loss maps of ZnO/ZnCr_2_O_4_ interface using (**a**) O-*K* and (**b**) Cr-*L*_2,3_ edges. ZnCr_2_O_4_ and ZnO are located at the top and bottom, respectively. The scan position of EELS profiles in (**c**) is indicated by the white arrow in (**a**). (**c**) EELS profiles of O-*K* and Cr-*L*_2,3_ edges across the interface. The distance between each spectrum is 1.6 nm. Close-ups of (**d**) the O-*K* edge at the two phases and the interface position, and (**e**) the Cr-*L2,3* edge at the ZnCr2O4 phase and the interface.

The core-loss EELS spectra of O and Cr obtained across a sharp ZnO/ZnCr_2_O_4_ interface are illustrated in Figure 4. It can be seen from Figure 4a that the O-*K* edge was detected in both ZnO and ZnCr_2_O_4_, with similar intensity in ZnCr_2_O_4_. In Figure 4b, the Cr-*L*_2,3_ edge was high in intensity at the ZnCr_2_O_4_ phase, but this signal dropped abruptly across the sharp interfacial region, and disappeared when it entered ZnO. This confirms the non-existence of other phases, as discussed previously. It is well established that the EELS O-*K* and Zn-*L*_2,3_ edges probe the unoccupied O 2*p* and Zn 3*d* states, respectively. We observed significant differences between ZnO and ZnCr_2_O_4_ as shown in Figure 4c, indicative of the different bonding schemes in the two materials. For the O-*K* edge, there was only one peak observed in the ZnO matrix, in accordance with previous measurements [38,39,40], while an obvious splitting occurred in ZnCr_2_O_4_, agreeing well with earlier reports [41,42,43], as also illustrated in Figure 4d. This was because in the ZnO unit cell, there was only one tetrahedron, which was composed of Zn and its four surrounding O, and each Zn-O bond exhibited a length of 1.98 Å. In comparison, both tetrahedral and octahedral configurations existed in the ZnCr_2_O_4_ unit cell. One Zn and its four surrounding O constitute a tetrahedron, while one Cr and its six surrounding O form an octahedron. The bond lengths of Zn-O and Cr-O were 2.04 Å and 1.95 Å, respectively. Furthermore, the O-*K* edge shifted from one peak to two peaks across the interface region. At the same time, the intensity of the Cr-*L*_2,3_ edge reduced progressively from ZnCr_2_O_4_ to ZnO at the interfacial region and disappeared when the probe was moved into the ZnO matrix. The oxidation states of the transition metal oxides to a large extent determine their physiochemical properties. ELNES analysis of the EELS spectrum, with its special valence sensitivity to 3*d* transition metals [44,45,46], is capable of revealing the electronic structure of target atoms (i.e., valence state, atomic coordination, and spin state). For instance, making use of the white line ratio (*L*_3_/*L*_2_) of the Cr-*L*_2,3_ edge, a number of studies have successfully analyzed the oxidation state in chromium oxides [42,43,47]. According to our experiments, the *L*_3_/*L*_2_ ratio remained at approximately 1.6 across the interface, indicative of no valence change for Cr. This was confirmed by the high similarity of the normalized intensities of *L*_3_ and *L*_2_ edges between the ZnCr_2_O_4_ phase and the interface position (Figure 4e).

**Figure 5:**
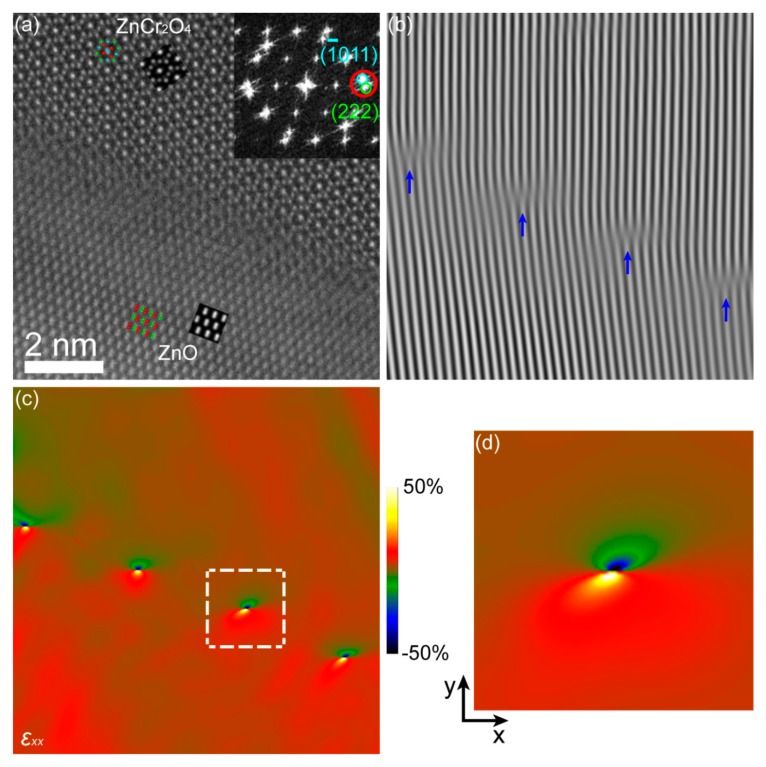
(**a**) High-angle annular dark-field (HAADF) image of ZnO [2$\bar{1}\bar{1}$3]/ZnCr_2_O_4_ [1$\bar{1}$0] interface. The inset at the upper right of the HAADF image displays its fast Fourier transform (FFT), and the circles show the lattice fringes. The simulated images and the corresponding projected models of ZnO and ZnCr_2_O_4_ are also inserted, and their close-ups are displayed in Figure S3b,c (Supplementary Materials). (**b**) Fourier-filtered image of ($\bar{1}$011)_ZnO_/(222)_ZnCr2O4_ lattice fringes with blue arrows indicating the dislocation lines. (**c**) Geometric phase analysis (GPA) *ε_xx_* maps. (**d**) Close-up of the white square in (**c**).

The structure and strain of some sharp ZnO/ZnCr_2_O_4_ interfaces (similar to the interface “*S*” as marked in Figure 2a) were found and investigated in this work. Figure 5 presents a typical sharp interface of ZnO [2$\bar{1}\bar{1}$3]/ZnCr_2_O_4_ [1$\bar{1}$0]. This interface area is indicated in Figure S2 (Supplementary Materials). The size of the HAADF image in Figure 5a is about 9.7 nm × 9.7 nm. The interface abruptness can be seen in Figure 5a. In order to clearly reveal the structure, the ABF image, as displayed in Figure S3a (Supplementary Materials), was recorded simultaneously with the HAADF image. There were dislocations at the lattice mismatched interfacial region, as indicated in the Fourier-filtered image produced with the ($\bar{1}$011)_ZnO_/(222)_ZnCr2O4_ lattice fringes (Figure 5b). The d-spacings of the ($\bar{1}$011)_ZnO_ and (222)_ZnCr2O4_ planes were similar, but the nominal mismatch was enhanced to approximately 9% by the 9° angle adopted at the interface. The strain was localized at misfit dislocations with a spacing in line with the expected mismatch. The localization of the strain was quantified by GPA using the ($\bar{1}$011)_ZnO_/(222)_ZnCr2O4_ reflections, as displayed in Figure 5c. An unstrained region selected from homogeneous ZnCr_2_O_4_ was used as reference. A close-up of the white square in Figure 5c is given in Figure 5d showing the strain field of a positive edge dislocation with projected Burgers vector **b** = [222]_ZnCr2O4_.

**Figure 6:**
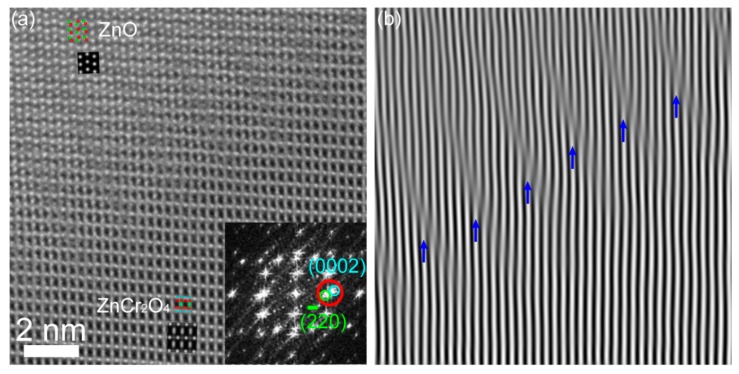
(**a**) HAADF image of ZnO [1$\bar{2}$10]/ZnCr_2_O_4_ [112] interface. The inset at the bottom right of the HAADF image displays its FFT, and the circles show the lattice fringes. The simulated images and the corresponding projected models of ZnO and ZnCr_2_O_4_ are also inserted, and their close-ups can be found in Figure S5b,c (Supplementary Materials). (**b**) Fourier-filtered image of (0002)_ZnO_/($\bar{2}$20)_ZnCr2O4_ lattice fringes.

HAADF imaging was also used to investigate ZnO/ZnCr_2_O_4_ interfaces in projection such as the interface “*N*” shown in Figure 2a. A representative example is given in Figure 6, which illustrates the atomically resolved HAADF image and its filtered image at the non-sharp interface of ZnO [1$\bar{2}$10]/ZnCr_2_O_4_ [112] in a non-edge on orientation. An overview HAADF image of the particle and the positions of the interface region can be found in Figure S4 (Supplementary Materials). The HAADF image size in Figure 6a is approximately 12.5 nm × 12.5 nm. The ABF image, which was acquired simultaneously with the HAADF image, is given in Figure S5a (Supplementary Materials) as a support for the structure determination. The Fourier-filtered image of (0002)_ZnO_/($\bar{2}$20)_ZnCr2O4_ lattice fringes in Figure 6b evidences the existence of parallel dislocations running at the interface. The angle difference between the (0002)_ZnO_ and ($\bar{2}$20)_ZnCr2O4_ planes was negligible, but there was a large lattice mismatch, and the d-spacings were 2.6 Å and 2.94 Å, respectively. This resulted in several misfit dislocations, as marked by blue arrows in Figure 6b that ensured the semi-coherency of the interface but imply chemical bond rearrangement. In general, more continuity at an interface leads to less rearrangement and reduced interfacial energy [48]. Other interfaces have been studied as well (not shown), and the strain deduced accordingly. However, dislocations remain a key parameter for the introduction of strain in the ZnO/ZnCr_2_O_4_ heterojunction interface.

## 4. Conclusions

We combined probe-corrected STEM, (monochromatic) EELS, EDX, and GPA to investigate the structure and strain of nano ZnCr_2_O_4_ inclusions in a ZnO matrix. The defects and their associated strain fields at ZnO/ZnCr_2_O_4_ interfaces were investigated by means of atomic-resolution STEM imaging and GPA analysis. The ZnO [2$\bar{1}\bar{1}$3]/ZnCr_2_O_4_ [1$\bar{1}$0] interface was found to be sharp with a low dislocation density, while the ZnO [1$\bar{2}$10]/ZnCr_2_O_4_ [112] interface showed a large lattice mismatch and the introduction of high dislocation densities. Thus, the ZnO [2$\bar{1}\bar{1}$3]/ZnCr_2_O_4_ [1$\bar{1}$0] interface provides a promising starting point for applications that rely on a defect-free interface which promotes efficient electrical transport without a high degree of charge carrier recombination through defect states. Furthermore, the band gap of the embedded ZnCr_2_O_4_ particle was mapped out with high spatial resolution utilizing the nanoscale resolving power of monochromated EELS in conjunction with probe-corrected STEM. The bandgap was found to be 3.84 ± 0.03 eV, which is in line with bulk values of ZnCr_2_O_4_ and in combination with the EDX and core-loss EELS results confirmed the formation of a phase-pure ZnCr_2_O_4_ particle embedded in ZnO. This provides the foundation for future studies of band-gap confinement effects in embedded ZnCr_2_O_4_ nanocrystals.

## Supplementary Materials

The following are available online at https://www.mdpi.com/1996-1944/12/6/888/s1, Figure S1: (a) HAADF and (b) ABF images of ZnO viewed from the [0001] orientation. (c) HAADF and (d) ABF imaging of ZnO observed from the [10$\bar{1}$0] direction. (e) HAADF and (f) ABF images of ZnCr_2_O_4_ viewed from the [100] orientation. (g) HAADF and (h) ABF imaging of ZnCr_2_O_4_ observed from the [111] direction. The insets show the model and simulated image. The green, cyan, and red balls represent Zn, Cr, and O, respectively. Figure S2: HAADF image of ZnCr_2_O_4_ nanoparticle in ZnO matrix. The red arrow indicates the interface area as analyzed in Figure 5 (main text) and Figure S3. Figure S3: (a) ABF image of ZnO [2$\bar{1}\bar{1}$3]/ZnCr_2_O_4_ [1$\bar{1}$0] interface, observed simultaneously with the HAADF image in Figure 5a (main text). The insets show the projected atomic models and simulated images. Close-ups of the experimental HAADF and simulated images as well as models of (b) ZnCr_2_O_4_ and (c) ZnO. The green, cyan and red balls represent Zn, Cr and O, respectively. Figure S4: HAADF image of ZnCr_2_O_4_ nanoparticle in ZnO matrix. The red arrow shows the interface region as investigated in Figure 6 (main text) and Figure S5. Figure S5: (a) ABF image of ZnO [1$\bar{2}$10]/ZnCr_2_O_4_ [112] interface, taken simultaneously with the HAADF image in Figure 6a (main text). The insets show the projected atomic models and simulated images. Close-ups of the experimental HAADF and simulated images as well as models of (b) ZnO and (c) ZnCr_2_O_4_. The green, cyan and red balls represent Zn, Cr and O, respectively. Table S1: Unit cell parameter of ZnO from XRD experiment and literature. *α* = *β* = 90°, *γ* = 120°. Table S2: Unit cell parameter of ZnCr_2_O_4_ from XRD experiment and literature. *α* = *β* = *γ* =90°.

## References

[1] Choudhary P., Yadav A., Varshney D. (2017). Structural and optical studies of nanocrystalline ZnCr_2_O_4_ and CoCr_2_O_4_ spinel. AIP Conf. Proc..

[2] Gene S.A., Saion E., Shaari A.H., Kamarudin M.A., Al-Hada N.M., Kharazmi A. (2014). Structural, optical, and magnetic characterization of spinel zinc chromite nanocrystallines synthesised by thermal treatment method. J. Nanomater..

[3] Niu X., Du W., Du W. (2004). Preparation and gas sensing properties of ZnM_2_O_4_ (M = Fe, Co, Cr). Sens. Actuators B Chem..

[4] Bayhan M., Hashemi T., Brinkman A.W. (1997). Sintering and humidity-sensitive behaviour of the ZnCr_2_O_4_–K_22_CrO_4_ ceramic system. J. Mater. Sci..

[5] Yokomizo Y., Uno S., Harata M., Hiraki H., Yuki K. (1983). Microstructure and humidity-sensitive properties of ZnCr_2_O_4_-LiZnVO_4_ ceramic sensors. Sens. Actuators.

[6] Gabr R.M., Girgis M.M., El-Awad A.M. (1992). Formation, conductivity and activity of zinc chromite catalyst. Mater. Chem. Phys..

[7] El-Sharkawy E.A. (1998). Textural, Structural and Catalytic Properties of ZnCr_2_O_4_–A_12_O_3_ Ternary Solid Catalysts. Adsorpt. Sci. Technol..

[8] Mousavi Z., Soofivand F., Esmaeili-Zare M., Salavati-Niasari M., Bagheri S. (2016). ZnCr_2_O_4_ Nanoparticles: Facile Synthesis, Characterization, and Photocatalytic Properties. Sci. Rep..

[9] Rajadurai S. (1987). Synthesis, structural characterization and catalytic study of ZnCrFeO_4_ spinel. Mater. Chem. Phys..

[10] Lee S.H., Broholm C., Ratcliff W., Gasparovic G., Huang Q., Kim T.H., Cheong S.W. (2002). Emergent excitations in a geometrically frustrated magnet. Nature.

[11] Vankudoth K., Padmasri A.H., Sarkari R., Velisoju V.K., Gutta N., Sathu N.K., Rohita C.N., Akula V. (2017). The role of Lewis acid-base pair sites in ZnO-ZnCr_2_O_4_ catalysts for cyclization via dehydrogenative condensation of crude glycerol and 1,2-propanediamine for the synthesis of 2,6-dimethylpyrazine. New J. Chem..

[12] Thennarasu G., Sivasamy A. (2015). Synthesis and characterization of nanolayered ZnO/ZnCr_2_O_4_ metal oxide composites and its photocatalytic activity under visible light irradiation. J. Chem. Technol. Biotechnol..

[13] Liang Y.C., Hsia H.Y., Cheng Y.R., Lee C.M., Liu S.L., Lin T.Y., Chung C.C. (2015). Crystalline quality-dependent gas detection behaviors of zinc oxide-zinc chromite p-n heterostructures. CrystEngComm.

[14] Song H., Laudenschleger D., Carey J.J., Ruland H., Nolan M., Muhler M. (2017). Spinel-Structured ZnCr_2_O_4_ with Excess Zn Is the Active ZnO/Cr2O3 Catalyst for High-Temperature Methanol Synthesis. ACS Catal..

[15] Pokhrel S., Jeyaraj B., Nagaraja K.S. (2003). Humidity-sensing properties of ZnCr_2_O_4_–ZnO composites. Mater. Lett..

[16] David B., Williams C.B.C. (2009). Transmission Electron Microscopy. A Textbook for Materials Science.

[17] LeBeau J.M., Findlay S.D., Allen L.J., Stemmer S. (2008). Quantitative Atomic Resolution Scanning Transmission Electron Microscopy. Phys. Rev. Lett..

[18] Tang Y.L., Zhu Y.L., Ma X.L. (2016). On the benefit of aberration-corrected HAADF-STEM for strain determination and its application to tailoring ferroelectric domain patterns. Ultramicroscopy.

[19] Zhu Y., Ophus C., Ciston J., Wang H. (2013). Interface lattice displacement measurement to 1pm by geometric phase analysis on aberration-corrected HAADF STEM images. Acta Mater..

[20] Couillard M., Radtke G., Botton G.A. (2013). Strain fields around dislocation arrays in a Σ9 silicon bicrystal measured by scanning transmission electron microscopy. Philos. Mag..

[21] Cooper D., Royer C.L., Béché A., Rouvière J.L. (2012). Strain mapping for the silicon-on-insulator generation of semiconductor devices by high-angle annular dark field scanning electron transmission microscopy. Appl. Phys. Lett..

[22] Trindade T., O’Brien P., Pickett N.L. (2001). Nanocrystalline Semiconductors: Synthesis, Properties, and Perspectives. Chem. Mater..

[23] Regulacio M.D., Han M.Y. (2010). Composition-Tunable Alloyed Semiconductor Nanocrystals. Acc. Chem. Res..

[24] Bailey R.E., Nie S. (2003). Alloyed Semiconductor Quantum Dots: Tuning the Optical Properties without Changing the Particle Size. J. Am. Chem. Soc..

[25] Brink H.A., Barfels M.M.G., Burgner R.P., Edwards B.N. (2003). A sub-50 meVspectrometer and energy filter for use in combination with 200 kV monochromated (S)TEMs. Ultramicroscopy.

[26] Erni R., Browning N.D. (2005). Valence electron energy-loss spectroscopy in monochromated scanning transmission electron microscopy. Ultramicroscopy.

[27] Bosman M., Tang L.J., Ye J.D., Tan S.T., Zhang Y., Keast V.J. (2009). Nanoscale band gap spectroscopy on ZnO and GaN-based compounds with a monochromated electron microscope. Appl. Phys. Lett..

[28] Zhan W., Granerød C.S., Venkatachalapathy V., Johansen K.M.H., Jensen I.J.T., Kuznetsov A.Y., Prytz Ø. (2017). Nanoscale mapping of optical band gaps using monochromated electron energy loss spectroscopy. Nanotechnology.

[29] QSTEM: Quantitative TEM/STEM Simulations. http://qstem.org/.

[30] Egerton R.F. (2007). Limits to the spatial, energy and momentum resolution of electron energy-loss spectroscopy. Ultramicroscopy.

[31] Granerød C.S., Zhan W., Prytz Ø. (2018). Automated approaches for band gap mapping in STEM-EELS. Ultramicroscopy.

[32] Zhan W., Venkatachalapathy V., Aarholt T., Kuznetsov A.Y., Prytz Ø. (2018). Band gap maps beyond the delocalization limit: Correlation between optical band gaps and plasmon energies at the nanoscale. Sci. Rep..

[33] Hytch M.J., Putaux J.L., Penisson J.M. (2003). Measurement of the displacement field of dislocations to 0.03 Å by electron microscopy. Nature.

[34] Hÿtch M.J., Putaux J.L., Thibault J. (2006). Stress and strain around grain-boundary dislocations measured by high-resolution electron microscopy. Philos. Mag..

[35] Johnson C.L., Snoeck E., Ezcurdia M., Rodriguez-Gonzalez B., Pastoriza-Santos I., Liz-Marzan L.M., Hytch M.J. (2008). Effects of elastic anisotropy on strain distributions in decahedral gold nanoparticles. Nat. Mater..

[36] Francis S., Saravanan R., Berchmans L.J. (2013). Phase analysis in Zn_1−x_ Cr_x_ O through charge density. Phase Transit..

[37] Fu C.F., Han L.F., Liu C., Gao Y.F. (2013). Effects of Cr-doping concentration on the structural, optical, and magnetic properties of ZnO thin films. Phys. Status Solidi.

[38] Quang H.T., Bachmatiuk A., Dianat A., Ortmann F., Zhao J., Warner J.H., Eckert J., Cunniberti G., Rümmeli M.H. (2015). In Situ Observations of Free-Standing Graphene-like Mono- and Bilayer ZnO Membranes. ACS Nano.

[39] Ding Y., Wang Z.L. (2005). Electron energy-loss spectroscopy study of ZnO nanobelts. J. Electron Microsc..

[40] Wang N., Yang Y., Yang G. (2011). Great blue-shift of luminescence of ZnO nanoparticle array constructed from ZnO quantum dots. Nanoscale Res. Lett..

[41] Eustace D.A., McComb D.W., Craven A.J. (2010). Probing magnetic order in EELS of chromite spinels using both multiple scattering (FEFF8.2) and DFT (WIEN2k). Micron.

[42] Arévalo-López Á.M., Alario-Franco M.Á. (2009). Reliable Method for Determining the Oxidation State in Chromium Oxides. Inorg. Chem..

[43] Daulton T.L., Little B.J. (2006). Determination of chromium valence over the range Cr(0)–Cr(VI) by electron energy loss spectroscopy. Ultramicroscopy.

[44] Schmid H., Mader W. (2009). Distribution of Fe and In Dopants in Zinc Oxide: Combined EELS and EDS Analysis. Microsc. Anal..

[45] Prytz Ø., Taftø J., Ahn C.C., Fultz B. (2007). Transition metal d-band occupancy in skutterudites studied by electron energy-loss spectroscopy. Phys. Rev. B.

[46] Prytz Ø., Flage-Larsen E., Gu L., Sigle W., van Aken P.A., Taftø J. (2012). The charge-ordered spinel AlV2O4: High energy resolution EELS and computational studies. Phys. Rev. B.

[47] Daulton T.L., Little B.J., Lowe K., Jones-Meehan J. (2002). Electron energy loss spectroscopy techniques for the study of microbial chromium(VI) reduction. J. Microbiol. Methods.

[48] Sutton A.P., Balluffi R.W. (1995). Interfaces in Crystalline Materials.

