# Supplementary material for: ZnCr2O4 Inclusions in ZnO Matrix Investigated by Probe-Corrected STEM-EELS
Source: Materials (Basel). 2019 Mar 16;12(6):888. doi: 10.3390/ma12060888 (PMC6471317; doi:10.3390/ma12060888)
Supplement: Supplementary file 1 [file materials-12-00888-s001.pdf]

materials

MDPI

Supplementary Materials

$ZnCr_2O_4$ Inclusions in ZnO Matrix Investigated by Probe-Corrected STEM-EELS

Wei Zhan [1], Andrey Yurievich Kosinskiy [1], Lasse Vines [1], Klaus Magnus Johansen [1], Patricia Almeida Carvalho [2] and Øystein Prytz [1,*]

[1] Department of Physics, Centre for Materials Science and Nanotechnology, University of Oslo, N-0316 Oslo, Norway; zhanwei2009@163.com (W.Z.); andrey.kosinskiy@ntnu.no (A.Y.K.); lasse.vines@fys.uio.no (L.V.); k.m.h.johansen@fys.uio.no (K.M.J.)

[2] SINTEF Materials and Chemistry, NO-0314 Oslo, Norway; patricia.carvalho@sintef.no

* Correspondence: oystein.prytz@fys.uio.no

1. Unit Cell Parameters of ZnO and $ZnCr_2O_4$

Table S1. Unit cell parameter of ZnO from XRD experiment and literature. $\alpha = \beta = 90°$, $\gamma = 120°$.

Parameter	XRD (Å)	Literature (Å) [1]
$a = b$	3.2505(4)	3.2555(2)
c	5.2059(6)	5.2152(3)

Table S2. Unit cell parameter of $ZnCr_2O_4$ from XRD experiment and literature. $\alpha = \beta = \gamma = 90°$.

Parameter	XRD (Å)	Literature (Å) [2]
$a = b = c$	8.3293(9)	8.32765(8)

2. Atomic-Resolution Images of ZnO and $ZnCr_2O_4$

ZnO was directly observed by simultaneous high-resolution HAADF and ABF imaging in two low-index zone axes. It is evident from the [0001] orientation in Figure S1a-b that all the columns form a two-dimensional hexagonal structure, and each column contains both Zn and O. According to STEM images viewed from the $[10\bar{1}0]$ direction in Figure S1c-d, four Zn columns form a rectangle structure. In addition, we also imaged $ZnCr_2O_4$ with atomic resolution in two low-index zone axes. As can be seen from the [100] projection in Figure S1e-f, four CrO_2 atomic columns form a square in the HAADF and ABF images. For every two squares, there is one Zn column sitting in the square center. In the [111] zone axis as illustrated in Figure S1g-h, one central and six vertex Zn_2CrO_2 atomic columns form a hexagonal unit structure. A CrO_2 column exists between the two closest Zn_2CrO_2 columns. The Zn_2CrO_2 and CrO_2 columns display remarkably different contrast. Not unexpected, in the HAADF image, Zn_2CrO_2 column is much brighter than CrO_2 column. The Zn_2CrO_2 column is significantly darker than CrO_2 column in the ABF image.

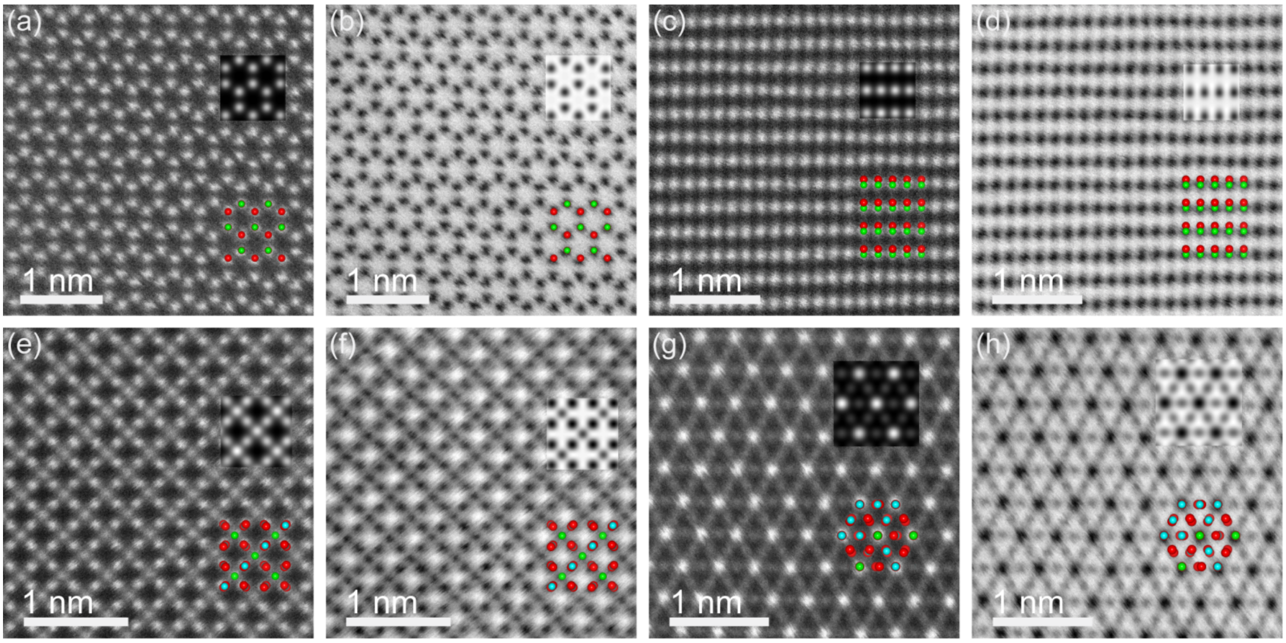

Figure S1. (**a**) HAADF and (**b**) ABF images of ZnO viewed from the [0001] orientation. (**c**) HAADF and (**d**) ABF imaging of ZnO observed from the [10$\bar{1}$0] direction. (**e**) HAADF and (**f**) ABF images of $ZnCr_2O_4$ viewed from the [100] orientation. (**g**) HAADF and (**h**) ABF imaging of $ZnCr_2O_4$ observed from the [111] direction. The insets show the model and simulated image. The green, cyan, and red balls represent Zn, Cr, and O, respectively.

3. ZnO/$ZnCr_2O_4$ Interfaces

The red arrow in Figure S2 points at the interface area shown in Figure 5 (main text) and Figure S3.

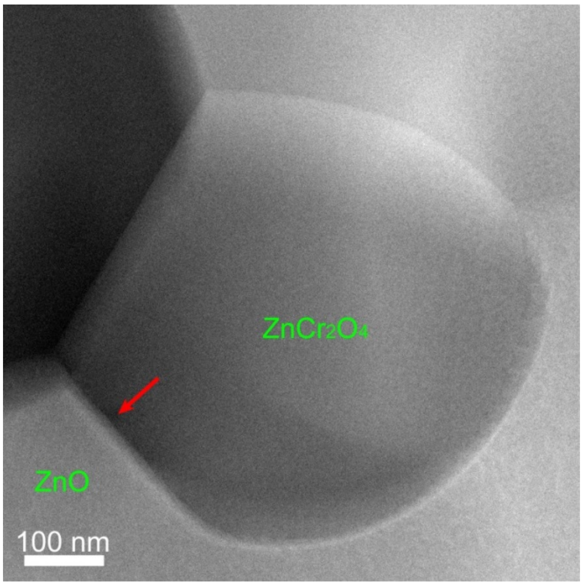

Figure S2. HAADF image of $ZnCr_2O_4$ nanoparticle in ZnO matrix. The red arrow indicates the interface area as analyzed in Figure 5 (main text) and Figure S3.

Figure S3a displays an ABF image of the ZnO [2$\bar{1}\bar{1}$3]/$ZnCr_2O_4$ [1$\bar{1}$0] interface, which was observed simultaneously with the HAADF image in Figure 5a (main text).

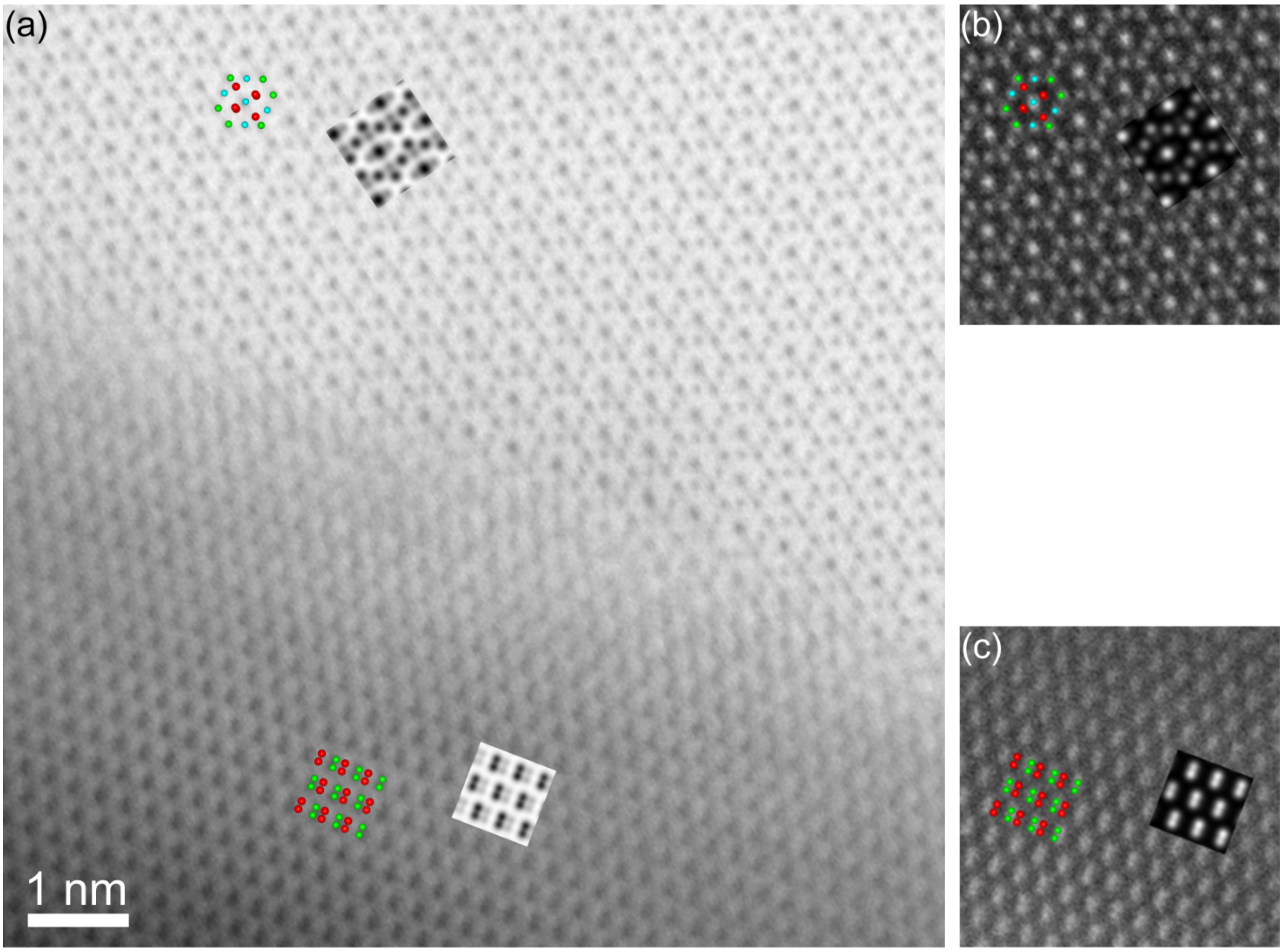

Figure S3. (**a**) ABF image of ZnO [$2\bar{1}\bar{1}3$]/$ZnCr_2O_4$ [$1\bar{1}0$] interface, observed simultaneously with the HAADF image in Figure 5a (main text). The insets show the projected atomic models and simulated images. Close-ups of the experimental HAADF and simulated images as well as models of (**b**) $ZnCr_2O_4$ and (**c**) ZnO. The green, cyan and red balls represent Zn, Cr and O, respectively.

The interface region in Figure 6 (main text) and Figure S5 is displayed by the red arrow in Figure S4.

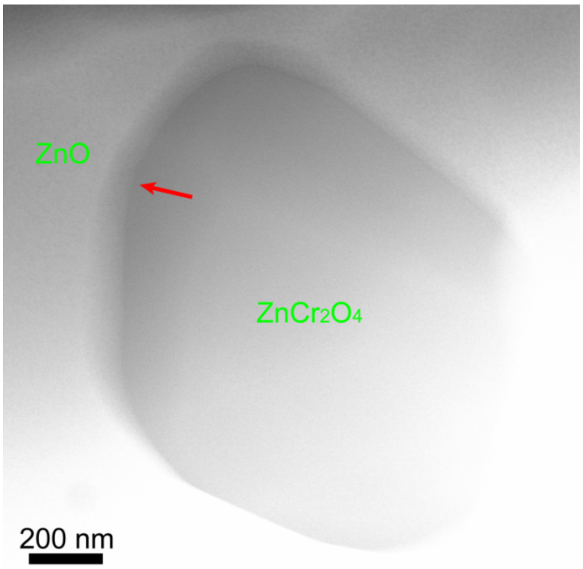

Figure S4. HAADF image of $ZnCr_2O_4$ nanoparticle in ZnO matrix. The red arrow shows the interface region as investigated in Figure 6 (main text) and Figure S5.

Figure S5a illustrates ABF image of the ZnO [1$\bar{2}$10]/$ZnCr_2O_4$ [112] interface, which was recorded simultaneously with the HAADF image in Figure 6a (main text).

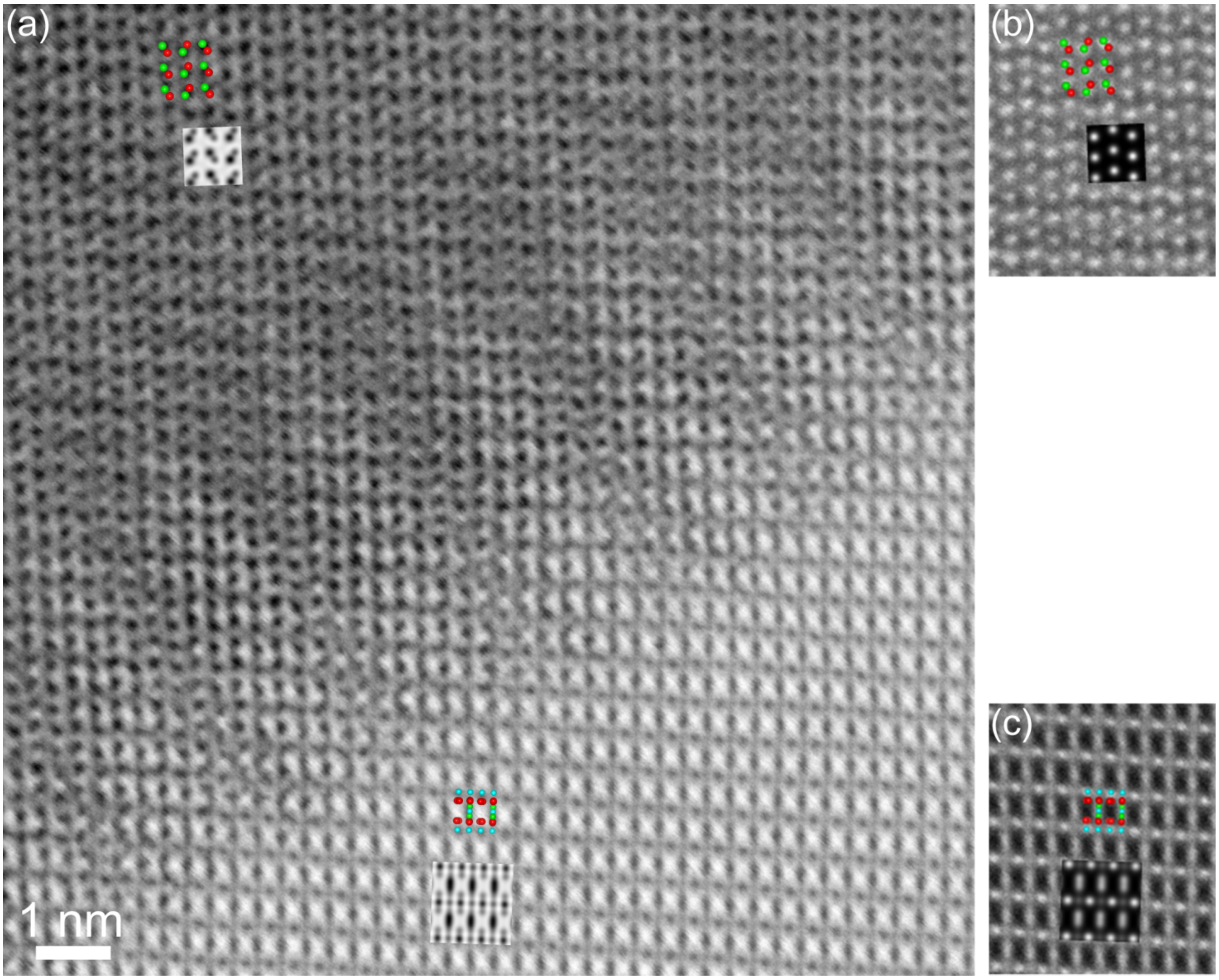

Figure S5. (**a**) ABF image of ZnO [1$\bar{2}$10]/$ZnCr_2O_4$ [112] interface, taken simultaneously with the HAADF image in Figure 6a (main text). The insets show the projected atomic models and simulated images. Close-ups of the experimental HAADF and simulated images as well as models of (**b**) ZnO and (**c**) $ZnCr_2O_4$. The green, cyan and red balls represent Zn, Cr and O, respectively.

1. Francis, S., R. Saravanan, and L.J. Berchmans, *Phase analysis in $Zn_{1-x}Cr_xO$ through charge density.* Phase Transitions, 2013. **86**(6): p. 620-632.
2. Moureen, C.K., et al., *Crystal structures of spin-Jahn–Teller-ordered $MgCr_2O_4$ and $ZnCr_2O_4$.* Journal of Physics: Condensed Matter, 2013. **25**(32): p. 326001.

© 2019 by the authors. Submitted for possible open access publication under the terms and conditions of the Creative Commons Attribution (CC BY) license (http://creativecommons.org/licenses/by/4.0/).
